# Individual Differences in the Association Between Celebrity Worship and Subjective Well-Being: The Moderating Role of Gender and Age

**DOI:** 10.3389/fpsyg.2021.651067

**Published:** 2021-05-14

**Authors:** Ágnes Zsila, Gábor Orosz, Lynn E. McCutcheon, Zsolt Demetrovics

**Affiliations:** ^1^Institute of Psychology, Pázmány Péter Catholic University, Budapest, Hungary; ^2^Unité de Recherche Pluridisciplinaire Sport Santé Société, Laboratoire Sherpas, Université d’Artois, Liévin, France; ^3^North American Journal of Psychology, Winter Garden, FL, United States; ^4^Centre of Excellence in Responsible Gaming, University of Gibraltar, Gibraltar, Gibraltar; ^5^Institute of Psychology, Eötvös Loránd University, Budapest, Hungary

**Keywords:** celebrity worship, daytime sleepiness, moderation analysis, self-esteem, subjective well-being

## Abstract

The association of celebrity worship with mental health concerns has been extensively studied in the past two decades. However, there is a lack of research on basic demographic characteristics that can potentially alter the link between celebrity admiration and different aspects of mental health. The present study investigates the possible moderating role of gender, age, and opposite/same-gender celebrity selection on the association of celebrity worship with general well-being, self-esteem and perceived daytime sleepiness. A total of 1763 Hungarian adults (66.42% men, *M_*age*_* = 37.2 years, *SD* = 11.4) completed an online survey focusing on attitudes and behaviors relating to celebrities and mental well-being. The moderation analysis showed that (i) the negative association between celebrity worship and self-esteem was slightly stronger for women than for men, and (ii) the association between celebrity worship and perceived daytime sleepiness was slightly stronger for younger individuals than for older ones. Although both gender and age were particularly weak moderators, these results draw the attention to some potential individual differences when interpreting links between celebrity worship and different aspects of mental health.

## Introduction

The influential role of celebrities on mass media consumers’ attitudes and behavior has generated considerable research in the past decades (Fraser and Brown, 2002; Dix et al., 2010; Nisbett and DeWalt, 2016). A growing body of research has focused on the psychological characteristics of fans with an excessive admiration toward a celebrity (i.e., “celebrity worshippers”) (McCutcheon et al., 2004; Brooks, 2018). Besides major demographic characteristics, several studies have investigated the association between celebrity worship and well-being (see Sansone and Sansone, 2014 for a review). These studies have found weak but consistent associations between high celebrity worship and indicators of poor mental health such as depression, anxiety, somatic symptoms (Maltby et al., 2001), impulsivity (McCutcheon et al., 2014; Aruguete et al., 2020), lower levels of life satisfaction (Maltby et al., 2004; Aruguete et al., 2019b), relative deprivation (Aruguete et al., 2020), lower self-esteem (Reeves et al., 2012), higher levels of psychological distress (Reyes et al., 2016), dissociative experiences (Maltby et al., 2006), and maladaptive daydreaming (Zsila et al., 2019). Despite the considerable research interest in the psychological well-being of individuals who admire a celebrity, still little is known about whether there are any specific individual differences between fans that can possibly amplify or mitigate their vulnerability to mental health problems. Some recent multidisciplinary studies have found evidence that media attention toward a celebrity’s mental illness can raise public awareness of certain mental disorders, which in turn can encourage fans to seek professional help (Hoffman and Tan, 2015; Lee, 2018, 2019). However, substantial differences were found in the prevalence of mental health concerns and help-seeking attitudes in terms of gender and age, suggesting that women are more likely to experience internalizing mental disorders such as depression and anxiety, while older individuals are more likely to seek psychological help (e.g., Mackenzie et al., 2006; Rosenfield and Mouzon, 2013). Drawing on previous findings on the association of celebrity worship with demographic characteristics and psychological well-being, the present study investigates the potential moderating role of basic demographic variables such as gender, age and opposite/same-gender celebrity worship on the association between celebrity worship and three relevant indicators of subjective well-being (i.e., general well-being, self-esteem, and perceived daytime sleepiness). This study is among the first that explores possible differences among fans in their mental health vulnerability based on major demographics. Prior research on celebrity worship and psychological health concerns were generally conducted on a limited number of undergraduates (200–400 participants), which could not allow for the investigation of possible individual differences in this association. This study endeavors to extend knowledge on the association between celebrity worship and well-being by investigating the possible moderating role of major demographics on this association, using a relatively large sample of fans. To the authors’ knowledge, this study employs the largest sample that has been used for celebrity worship studies so far. The exploration of individual differences in the association between celebrity worship and mental well-being could possibly contribute to a more nuanced understanding of the nature of this weak but consistent relationship. Furthermore, exploring individual differences in this association can help identifying some specific subgroups of fans that may need particular attention in mental health care. Based upon these findings, more targeted and effective strategies could possibly be offered in an effort to reduce the harms associated with an excessive admiration toward a favorite celebrity in some specific subgroups.

### Celebrity Worship

Celebrity worship is a multidimensional construct defined as an obsessive, one-sided emotional attachment to a celebrity (McCutcheon et al., 2002). According to the theoretical model proposed by McCutcheon et al. (2002), celebrity worship can be interpreted as a continuum ranging from healthy enthusiasm (i.e., the fan enjoys being with others who like his/her favorite celebrity) to more obsessive feelings (i.e., the fan considers a celebrity to be his/her soul mate) and behaviors (i.e., if the favorite celebrity asked the fan to do something illegal, he/she would do it).

Findings concerning gender differences in celebrity worship levels are inconsistent (Brooks, 2018). Some studies reported women expressing higher celebrity worship than men (e.g., Swami et al., 2011; Huh, 2012), while other studies found no gender difference (e.g., Ashe et al., 2005; Maltby and Day, 2011). In addition, a few studies reported higher celebrity worship levels among men (North et al., 2005; McCutcheon et al., 2016a).

With regard to age, studies consistently indicated that celebrity worship levels slightly decline with age (see Brooks, 2018 for a review). Lin and Lin (2007) suggested that idol worship is most prevalent among adolescents, and youth who are fascinated by a celebrity of the opposite gender exhibit generally higher levels of admiration. Recent findings have indicated that women are more likely to select a favorite celebrity of the opposite gender than men (Greenwood et al., 2018; Collisson et al., 2020). Although Greenwood et al. (2018) reported higher levels of celebrity worship among university students who selected a favorite celebrity of the opposite gender, Collisson et al. (2020) found no difference in celebrity worship levels with regard to opposite- and same-gender celebrity worship among adults with a mean age of 39 years. Provided that the present study also employs an adult sample with a mean age of 37 years, it is assumed that no difference will be found in celebrity worship levels with regard to opposite- and same-gender celebrity selection. Furthermore, it is expected that opposite- and same-gender celebrity worship plays no significant role in the association between celebrity worship and subjective well-being.

### General Well-Being

Subjective well-being is an indicator of perceived long-term happiness, positive affect and life satisfaction (Diener, 2009). Although most studies reported a lack of gender difference in the level of subjective well-being (see Inglehart, 1990 for a review), Inglehart (2002) found evidence that gender differences can be observed in some specific age groups. Using a large-scale sample involving participants from 65 societies, Inglehart (2002) revealed that women under 45 years of age had higher levels of subjective well-being than men, while this difference could not be observed among individuals over 45 years of age. Despite that age was identified as an influential factor when investigating gender differences in subjective well-being, Kunzmann et al. (2000) found no causal relationship between age and subjective well-being. Drawing upon these findings, it can be hypothesized that the association between celebrity worship and general well-being will be stronger for women than for men, while age will not affect the strength of this association.

### Self-Esteem

Self-esteem reflects “the individual’s positive or negative attitude toward the self” (Rosenberg et al., 1995, p.141). A large amount of studies found evidence for the association between mental health problems and lowered self-esteem (e.g., Corrigan et al., 2006; Picco et al., 2016; Silvestri et al., 2018). Recent large-scale studies have confirmed previous findings demonstrating that women tend to report lower levels of self-esteem than men (Orth et al., 2010; Bleidorn et al., 2016). It was also found that self-esteem increases from adolescence to middle adulthood, and declines after 60 years of age (Orth et al., 2010). However, low self-esteem was consistently associated with poorer mental health at different stages of adulthood (Orth et al., 2009). Based upon these results, it can be hypothesized that the relationship between celebrity worship and self-esteem will be stronger for women than for men, while the strength of this association will be similar across the adult lifespan in the present sample.

### Perceived Daytime Sleepiness

Maladaptive daydreaming had a positive relationship with several psychological difficulties such as depressive symptoms, anxiety and obsessive-compulsive disorder (Somer et al., 2017). Maladaptive daydreaming was also associated with celebrity worship (Zsila et al., 2019), and frequent daydreaming had a positive relationship with daytime sleepiness (Carciofo et al., 2014). Daytime sleepiness is generally associated with feelings of being exhausted, weary or worn out (Marques et al., 2019) and impairs cognitive performance (Merlino et al., 2010). Previous research has demonstrated that women (Kim and Young, 2005; Marques et al., 2019) and younger individuals (Bixler et al., 2005; Kim and Young, 2005) experience more frequent daytime sleepiness compared to men and older individuals. Bixler et al. (2005) also found that the association between poor mental health and excessive daytime sleepiness was stronger for younger individuals. Therefore, it can be hypothesized that the association between celebrity worship and perceived daytime sleepiness will be stronger for women and younger individuals than for men and older individuals.

### Hypotheses

Although findings are mixed regarding gender differences in celebrity worship (Brooks, 2018), a recent study on a sample of Hungarian adults has reported that women exhibited slightly higher levels of celebrity worship than men (Zsila et al., 2019). The present research was also conducted using a Hungarian sample. Previous studies have also demonstrated that adult women are generally characterized by higher levels of subjective well-being (Inglehart, 2002), lower levels of self-esteem (Orth et al., 2010; Bleidorn et al., 2016), and more frequent feelings of daytime sleepiness (Kim and Young, 2005; Marques et al., 2019). Considering the substantial gender differences in all constructs explored in the present study, the following hypothesis is proposed:

H1: Gender will moderate the association of celebrity worship with general well-being, self-esteem, and perceived daytime sleepiness. Celebrity worship will be more strongly associated with general well-being, self-esteem, and perceived daytime sleepiness among women than among men.

Studies have indicated that celebrity worship levels slightly decrease with age (Brooks, 2018). However, age was not associated with either subjective well-being (Kunzmann et al., 2000) or self-esteem (Orth et al., 2009) among adults. By contrast, younger individuals were more likely to experience daytime sleepiness compared to older individuals (Kim and Young, 2005). Moreover, the relationship between poorer mental health and frequent daytime sleepiness was stronger for younger individuals (Bixler et al., 2005). Drawing upon this association, and provided that celebrity worship was associated with younger age and more frequent daydreaming experiences (Zsila et al., 2019), the following hypothesis is proposed:

H2: Age will not moderate the association of celebrity worship with general well-being and self-esteem, but will moderate the association between celebrity worship and perceived daytime sleepiness. Celebrity worship will be more strongly associated with perceived daytime sleepiness among younger individuals than among older individuals.

Although Lin and Lin (2007) indicated that younger individuals who admire a celebrity of the opposite gender tend to exhibit higher levels of admiration than older individuals and those with a same-gender favorite celebrity, a recent study by Collisson et al. (2020) found no difference in celebrity worship among middle-age adults with an opposite-gender favorite celebrity and those with a same-gender favorite celebrity. The mean age of the present sample (37 years) is similar to the sample used by Collisson et al. (2020) (39 years); therefore, no substantial difference is expected in celebrity worship levels with regard to opposite- or same-gender celebrity selection. Based upon this assumption, no significant difference is expected in the strength of relationship of celebrity worship with indicators of psychological well-being across opposite- and same-gender celebrity worship. Therefore, the following hypothesis is proposed:

H3: Opposite/same-gender celebrity worship will not moderate the association of celebrity worship with general well-being, self-esteem, and perceived daytime sleepiness.

## Materials and Methods

### Participants and Procedure

Participants were recruited from a popular Hungarian news website^1^, which also delivers news about public media figures. Provided that the present investigation focused on attitudes toward famous media personae, this website was considered as an appropriate platform for the data collection. Visitors of this website were considered being interested in the life and public appearance of famous people. Participants were invited to complete an online questionnaire focusing on attitudes toward celebrities and psychological well-being. Preceding the completion, participants were informed about the general aim of the study and were requested to provide informed consent by ticking a box if they were over 18 years of age and agreed to the terms and conditions. This study was approved by the Institutional Review Board of the research team’s university (protocol number: 2018/14-2) and was carried out in compliance with the Declaration of Helsinki.

A total of 1836 participants completed the total questionnaire (66.83% men, *M_*age*_* = 37.14 years, *SD* = 12.06, age ranged from 14 to 79 years). Underage respondents (*n* = 12) and individuals who did not provide information regarding their favorite celebrity (e.g., left the answer sheet blank or selected a family member or a fictitious character) were excluded from further data analysis (*n* = 61). Therefore, the final sample comprised 1763 adult participants (66.42% men, *M_*age*_* = 37.22 years, *SD* = 11.38, age ranged from 18 to 79 years). Major demographic characteristics of the sample are presented in Table 1.

**TABLE 1:** Demographic characteristics of the sample (*N* = 1763).

	Participants (*N* = 1763)
**Gender**	
Men *n* (%)	1171 (66.42%)
Women *n* (%)	592 (33.58%)
**Age**	
Age (years) *Mean (SD)*	37.22 (11.38)
**Educational level**	
Received primary education *n* (%)	22 (1.25%)
Secondary school certificate *n* (%)	497 (28.19%)
College degree or higher *n* (%)	1244 (70.56%)
**Current studies/work**	
Student *n* (%)	140 (7.94%)
Has a job *n* (%)	1185 (67.21%)
Student and has a job *n* (%)	322 (18.26%)
No current studies or a job *n* (%)	116 (6.58%)

The majority of participants earned a college degree or higher, while 28.19% of participants received a secondary school certification, and only a small proportion of respondents completed eight or less classes at primary school. The majority of participants had a full-time or a part-time job at the time of the data collection, while 18.26% of participants had a job and also studied, and a small proportion of participants were students who had no job. Furthermore, only a small minority of participants reported not having a job or current studies in progress.

### Measures

Besides demographic characteristics, participants’ attitudes toward their favorite celebrity, general well-being, self-esteem, and perceived daytime sleepiness were assessed. The items of the Daytime Sleepiness Perception Scale (DSPS-4) were translated and back-translated to ensure that the content of the items remained unchanged. For this purpose, two independent translators of the research team have translated the four items to Hungarian of which one translator had no experience with daytime sleepiness assessment. Back-translation was carried out by two native Hungarian individuals with English proficiency. The translation process was assisted by a native English-speaking person. Due to the simplicity of the items, no severe discrepancies were observed. The final version was created based on consensual agreement among the translators. Consensus was reached with the assistance of an undergraduate student who had neither previous experience with daytime sleepiness assessment, nor previous knowledge on the purpose of this investigation. The final, consensual version was used in the survey after 6–8 experts examined the scale and consensually ensured its usability in the final form.

#### Information Concerning Participants’ Favorite Celebrities

First, participants were introduced to the definition of a celebrity based on the general description suggested by McCutcheon et al. (2004). Therefore, celebrity was defined as a famous living person or one who died during the respondent’s lifetime. In the following step, participants were asked to name their favorite celebrity. Second, participants selected the primary field of expertise of their favorite celebrity based on the categories (i.e., Acting, Music, Author, Artist, Video-Making [e.g., vlogger, YouTuber], Radio/TV presenter, News, Science, Sports, Medicine, Modeling, Politics, Religion, Other) that were used in previous studies (e.g., Sheridan et al., 2007; Griffith et al., 2013; Zsila et al., 2018). Third, participants indicated how long they had been fans of their favorite celebrity (1 = “less than 1 year,” 2 = “1–2 years,” 3 = “3–5 years,” 4 = “more than 5 years”).

#### Celebrity Worship

Participants’ attraction toward their favorite celebrity was assessed by the 23-item Celebrity Attitude Scale (CAS) (McCutcheon et al., 2002, 2004). Cronbach’s alphas of the total CAS have ranged from 0.84 to 0.94 in previous studies (see Brooks, 2018 for a review). A Hungarian translation of the CAS was made and used in previous studies (Zsila et al., 2018, 2019, 2021). These studies demonstrated that the translated CAS had good reliability indices (Cronbach’s alpha was 0.87 for the Entertainment–Social subscale, 0.87 and 0.88 for the Intense–Personal subscale, and 0.62 and 0.64 for the Borderline–Pathological subscale). Cronbach’s alpha for the total CAS was 0.93. Therefore, this translation was used in the present research. Participants indicated their level of agreement with each statement using a five-point Likert scale (ranging from 1 = “strongly disagree” to 5 = “strongly agree”). Higher scores indicate higher levels of celebrity worship. A scale score is computed by summing the items. The CAS comprises three subscales: Entertainment–Social (10 items; e.g., “My friends and I like to discuss what my favorite celebrity has done”), Intense–Personal (nine items; e.g., “The successes of my favorite celebrity are my successes also”), and Borderline–Pathological (four items; e.g., “If I were lucky enough to meet my favorite celebrity, and he/she asked me to do something illegal as a favor, I would probably do it”). Cronbach’s alpha was 0.84 for the Entertainment–Social subscale and 0.83 for the Intense–Personal subscale in the present study. However, the reliability of the Borderline–Pathological subscale was below the level of acceptance (Cronbach’s alpha was 0.55), similar to some recent studies that have reported Cronbach’s alphas between 0.43 and 0.54 (e.g., McCutcheon et al., 2012, 2016b; Reeves et al., 2012). Griffith et al. (2013) investigated the test-retest and internal reliability of the CAS subscales and found that the Cronbach’s alpha of the Borderline–Pathological subscale was 0.59 at the first time of assessment, and 0.58 3 months later. Earlier and more recent studies have also calculated a total score of the CAS by adding all items (McCutcheon et al., 2004, 2014). Cronbach’s alphas of the total scale ranged from 0.84 to 0.94 in these studies (McCutcheon et al., 2004, 2014). For this reason, and due to the high inter-correlations among the CAS subscales (generally above *r* = 0.50) (Jung and Hwang, 2016), several recent studies have used only the total score of the CAS in the data analysis (e.g., McCutcheon et al., 2014; Jung and Hwang, 2016; Aruguete et al., 2019a; Zsila et al., 2019). Indeed, the three CAS subscales have often correlated highly with each other. For example, CAS Entertainment–Social correlated 0.74 and 0.77 with CAS Intense–Personal and CAS Borderline–Pathological in a recent study by Aruguete et al., 2019b. In the same study, CAS Intense–Personal correlated 0.75 with CAS Borderline–Pathological. Following the common practice, the total score of the CAS was used in the present study (Cronbach’s alpha was 0.91).

#### General Well-Being

Participants’ general, subjective well-being was assessed using the Hungarian version of the five-item WHO-5 Well-Being Index (WHO-5; Bech et al., 2003; Susánszky et al., 2006). The Hungarian WHO–5 is a unidimensional assessment instrument that consists of five items (e.g., “I felt active and vigorous”). Participants indicated the closest response option to how they felt during the past month using a four-point Likert scale (ranging from 0 = “not at all characteristic” to 3 = “very characteristic”). A total score was calculated by summing the items. Higher scores indicate higher levels of well-being reported by participants. Cronbach’s alpha was 0.84 in the study by Bech et al. (2003), 0.85 in the Hungarian adaptation by Susánszky et al. (2006), and 0.81 in the present study.

#### Self-Esteem

Participants’ self-esteem was assessed by the Hungarian version of the Rosenberg Self-Esteem Scale (RSES-HU; Rosenberg, 1986; Urbán et al., 2014). The RSES-HU is a unidimensional assessment instrument that comprises ten items (e.g., “I feel that I have a number of good qualities”). Participants rated each item using a four-point Likert scale (ranging from 1 = “strongly disagree” to 4 = “strongly agree”). A total score was calculated by summing all items. Higher scores indicate higher levels of general self-esteem. Cronbach’s alpha ranged between 0.77 and 0.88 (Rosenberg, 1986), the values were 0.87 and 0.86 in the Hungarian adaptation by Urbán et al. (2014), and 0.90 in the present study.

#### Perceived Daytime Sleepiness

Participants’ perceived daytime sleepiness was assessed using the Daytime Sleepiness Perception Scale (DSPS-4; Marques et al., 2019). The unidimensional DSPS-4 comprises four items (e.g., “During the day I feel that my performance is impaired by being sleepy”). Participants rated each item on a five-point Likert scale (ranging from 0 = “never” to 4 = “always”). The sum of all items constitutes the total score. Higher scores represent higher levels of perceived daytime sleepiness. Cronbach’s alpha was 0.71 in the study by Marques et al. (2019), and 0.88 in the present study.

### Statistical Analysis

Data analysis was performed using SPSS 21.0 (IBM SPSS Inc., Chicago, IL, United States) and PROCESS modeling tool for SPSS (Hayes, 2013).

Participants were categorized into two groups based upon whether their gender matched with the gender of their favorite celebrity. If participants’ gender was identical to the gender of their favorite celebrity (i.e., men with a male favorite celebrity or women with a female favorite celebrity), this matching was coded and labeled as 0 = “same-gender celebrity worship.” When participants’ gender was different from the gender of their favorite celebrity (i.e., men with a female favorite celebrity or women with a male favorite celebrity), this mismatching was coded and labeled as 1 = “opposite-gender celebrity worship.”

First, *t*-tests and χ^2^-tests were conducted to explore possible differences in study-relevant variables across gender and opposite/same-gender celebrity worship types. For these group comparisons, effect size indices were also estimated (Hedges’ *g*) with 0.2 being interpreted as a small effect, 0.5 as a medium effect, and 0.8 as a large effect (Cohen, 1988).

Provided that significant differences were found in some study-relevant variables across gender and opposite/same-gender celebrity worship, in the following step, partial correlations were conducted to explore the associations between continuous variables (i.e., age, celebrity worship, general well-being, self-esteem, perceived daytime sleepiness) while controlling for gender and opposite/same-gender celebrity worship.

In the final step, several moderation models were constructed to test the possible moderating effect of gender, age, and opposite/same-gender celebrity worship (moderators) on the association of celebrity worship (predictor variable) with general well-being, self-esteem, and perceived daytime sleepiness (outcome variables). In these models, 95% bias-corrected confidence intervals were applied based on 1000 bootstrapped samples. Gender, age, and opposite/same-gender celebrity worship were added as covariates when applicable.

## Results

### Descriptive Statistics for Interest in Celebrities

Descriptive statistics for the main characteristics of celebrity interest in the present sample are presented in Table 2. Favorite celebrities selected by participants showed a large variety; only three celebrities were named by more than 2% of respondents. According to these reports, the three most frequently selected favorite celebrities in the present sample were Keanu Reeves (2.61%), Elon Musk (2.61%), and David Bowie (2.27%). The most frequently selected categories for the primary field of expertise of a favorite celebrity were music (32.79%) and acting (22.12%). The vast majority of participants admired their favorite celebrity for more than 3 years (88.03%). The majority of participants selected a favorite celebrity of the same gender (70.56%). Specifically, the vast majority of men selected a male favorite celebrity (92.57% of male participants), while only a smaller proportion of women selected a female favorite celebrity (27.03% of women).

**TABLE 2:** Descriptive statistics for variables exploring the interest for a favorite celebrity in the present sample.

	Participants (*N* = 1763)
**Favorite celebrity**	
**The five most frequently selected FC**	
Keanu Reeves	46 (2.61%)
Elon Musk	46 (2.61%)
David Bowie	40 (2.27%)
Freddy Mercury	29 (1.64%)
Péter Esterházy	27 (1.53%)
**Field of expertise of FC**	
Acting *n* (%)	390 (22.12%)
Music *n* (%)	578 (32.79%)
Author *n* (%)	153 (8.68%)
Artist *n* (%)	38 (2.16%)
Video-Making (e.g., vlogger, YouTuber) *n* (%)	19 (1.08%)
Radio/TV presenter *n* (%)	39 (2.21%)
News *n* (%)	6 (0.34%)
Science *n* (%)	179 (10.15%)
Sports *n* (%)	157 (8.91%)
Medicine *n* (%)	8 (0.45%)
Modeling *n* (%)	4 (0.23%)
Politics *n* (%)	72 (4.08%)
Religion *n* (%)	26 (1.47%)
Other *n* (%)	94 (5.33%)
**Period of time being a fan of FC**	
Less than 1 year *n* (%)	49 (2.78%)
1–2 years *n* (%)	162 (9.19%)
3–5 years *n* (%)	298 (16.90%)
More than 5 years *n* (%)	1254 (71.13%)
**Opposite/same gender celebrity worship**	
Men selecting male FC *n* (%)	1084 (61.49%)
Men selecting female FC *n* (%)	87 (4.93%)
Women selecting female FC *n* (%)	160 (9.07%)
Women selecting male FC *n* (%)	432 (24.50%)

*FC, favorite celebrity.*

### Group Comparisons Across Gender and Opposite/Same-Gender Celebrity Worship Types

With regard to gender, significant difference was found between men and women in opposite/same-gender celebrity worship and self-esteem (see Table 3). Specifically, women were more likely to select a person of the opposite gender as a favorite celebrity (*n* = 432; 72.97%) compared to men (*n* = 87; 7.43%) (χ^2^ = 813.21, *p* < 0.001), and the difference was large (ρ = 0.68). Additionally, women had lower levels of self-esteem (*M* = 28.78, *SD* = 5.88) compared to men (*M* = 29.95, *SD* = 5.80) (*t* = 3.99, *p* < 0.001). The effect size was small (Hedges’ *g* = 0.20).

**TABLE 3:** Group comparisons across gender and opposite/same-gender celebrity worship in major demographics, celebrity worship, and indicators of subjective well-being.

		Gender			Opposite/same-gender celebrity worship		
	Total *N* = 1763	Men *n* = 1171	Women *n* = 592	t/χ^2^	Same-gender *n* = 1244	Opposite-gender *n* = 519	t/χ^2^
**Major demographics**							
Age (years) *Mean (SD)(range: 18–79)*	37.22 (11.38)	36.91 (11.15)	37.83 (13.09)	–1.47	36.29 (11.08)	39.45 (13.22)	–4.79***
**Indicators of celebrity worship**							
Opposite-gender *n* (%)	519 (29.44%)	87 (7.43%)	432 (72.97%)		–	–	
Same-gender *n* (%)	1244 (71.66%)	1084 (92.57%)	160 (27.03%)	813.21***	–	–	–
Celebrity worship *Mean (SD) (range: 23–112)*	43.39 (13.85)	43.40 (13.90)	43.36 (13.75)	0.07	43.00 (13.63)	44.32 (14.34)	-1.83
**Indicators of subjective well-being**							
General well-being *Mean (SD) (range: 0–15)*	8.61 (2.89)	8.61 (2.88)	8.60 (2.92)	0.17	8.61 (2.86)	8.60 (2.90)	0.10
Self-esteem *Mean (SD) (range: 11–40)*	29.56 (5.85)	29.95 (5.80)	28.78 (5.88)	3.99***	29.80 (5.81)	28.98 (5.92)	2.69**
Perceived daytime sleepiness *Mean (SD) (range: 0–16)*	4.97 (2.98)	4.95 (3.02)	5.02 (2.91)	–0.51	5.02 (3.04)	4.86 (2.82)	1.07

***p < 0.001; **p < 0.01.

χ^2^-tests were conducted for opposite/same-gender celebrity worship when comparing males with females, while independent-samples *t*-tests were performed for all other group comparisons.

Individuals who selected a person of the opposite gender as a favorite celebrity were older (*M* = 39.45, *SD* = 13.22) than individuals who selected a person of the same gender as a favorite celebrity (*M* = 36.29, *SD* = 11.08) (*t* = –4.79, *p* < 0.001). However, the difference was relatively small (Hedges’ *g* = 0.27). Furthermore, individuals who selected a person of the same gender as a favorite celebrity (*M* = 29.80, *SD* = 5.81) had higher levels of self-esteem compared to individuals who selected a person of the opposite gender as a favorite celebrity (*M* = 28.98, *SD* = 5.92) (*t* = 2.69, *p* = 0.007). However, the effect size was again small (Hedges’ *g* = 0.14).

### Partial Correlations Among Age, Celebrity Worship, and Indicators of Subjective Well-Being

Celebrity worship was negatively associated with general well-being (*r* = –0.07, *p* = 0.004) and self-esteem (*r* = –0.16, *p* < 0.001) (see Table 4). These results indicate that individuals with higher levels of celebrity worship had poorer general well-being and lower levels of self-esteem. Furthermore, celebrity worship was positively associated with perceived daytime sleepiness (*r* = 0.14, *p* < 0.001) and negatively with age (*r* = –0.13, *p* < 0.001). These results indicate that individuals with higher levels of celebrity worship experienced daytime sleepiness more frequently, and younger individuals expressed greater admiration toward a favorite celebrity than older individuals. However, these associations were generally weak. Perceived daytime sleepiness also decreased with age (*r* = –0.12, *p* < 0.001). By contrast, self-esteem increased with age (*r* = 0.23, *p* < 0.001). General well-being was not associated with participants’ age (*r* = 0.01, *p* = 0.65).

**TABLE 4:** Partial correlations among celebrity worship, indicators of subjective well-being, and age, while controlling for gender and opposite/same-gender celebrity worship (*N* = 1763).

	(1)	(2)	(3)	(4)
(1) Celebrity worship	—			
(2) General well-being	–0.07**	—		
(3) Self-esteem	–0.16***	0.53***	—	
(4) Perceived daytime sleepiness	0.14***	–0.33***	–0.26***	—
(5) Age	–0.13***	0.01	0.23***	–0.12***

***p < 0.001; **p < 0.01; *p < 0.05.

### Testing the Moderating Role of Gender, Age, and Opposite/Same-Gender Celebrity Worship on the Association Between Celebrity Worship and Indicators of Subjective Well-Being

**FIGURE 1:**
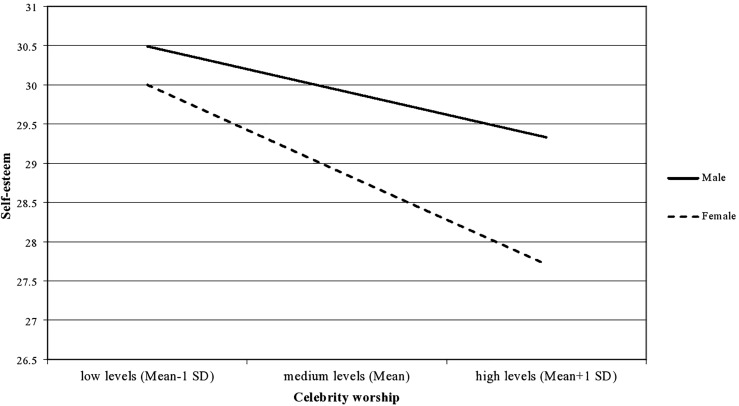
Gender differences in the association between celebrity worship and self-esteem.

In order to test the hypotheses, nine moderation models were constructed (see Table 5). Contrary to the first hypothesis, most associations between celebrity worship and indicators of subjective well-being were not moderated by gender (see Model 1a–c in Table 5). In more details, the moderating role of gender on the association of celebrity worship with general well-being (β = –0.02, *p* = 0.08) and perceived daytime sleepiness (β = 0.01, *p* = 0.35) was not supported by the data. However, the moderating role of gender on the association between celebrity worship and self-esteem received support as the interaction term was marginally significant (β = –0.04, *p* = 0.048). The negative association indicates that the relationship between celebrity worship and self-esteem is stronger for women than for men (see Figure 1). This result suggests that women with higher levels of celebrity worship had a higher tendency to report lowered self-esteem compared to men. However, the moderator effect of gender was relatively small, and the proportion of explained variance was also small (*R*^2^ = 8%). Overall, the first hypothesis was rejected.

**TABLE 5:** Moderation models testing the moderator role of gender, age, and opposite/same-gender celebrity worship on the association between celebrity worship and indicators of subjective well-being.

	β (95% C.I.)	*SE*	*p*	*R*^2^
Model 1a outcome variable: General well-being				0.01
Celebrity worship	0.01 (–0.02; 0.04)	0.01	0.51	
Gender	0.78 (–0.19; 1.76)	0.50	0.12	
Celebrity worship × Gender	–0.02 (–0.04; 0.002)	0.35	0.08	
Model 1b outcome variable: Self-esteem				0.08
Celebrity worship	–0.002 (–0.06; 0.06)	0.03	0.96	
Gender	0.71 (–1.19; 2.60)	0.97	0.46	
Celebrity worship × Gender	–0.04 (–0.08; <–0.001)	0.02	0.05	
Model 1c outcome variable: Perceived daytime sleepiness				0.03
Celebrity worship	0.01 (–0.02; 0.04)	0.02	0.33	
Gender	–0.10 (–1.09; 0.89)	0.50	0.85	
Celebrity worship x Gender	0.01 (–0.01; 0.03)	0.01	0.35	
Model 2a Outcome variable: General well-being				0.005
Celebrity worship	–0.02 (–0.05; 0.008)	0.02	0.15	
Age	–0.009 (–0.05; 0.03)	0.02	0.65	
Celebrity worship × Age	<0.001 (<–0.001; 0.001)	<0.001	0.61	
Model 2b outcome variable: Self-esteem				0.08
Celebrity worship	–0.09 (–0.15; –0.03)	0.03	0.003	
Age	0.07 (–0.005; 0.14)	0.04	0.07	
Celebrity worship × Age	<0.001 (<–0.001; 0.002)	<0.001	0.24	
Model 2c Outcome variable: Perceived daytime sleepiness				0.04
Celebrity worship	0.07 (0.03; 0.10)	0.02	<0.001	
Age	0.02 (–0.02; 0.06)	0.02	0.31	
Celebrity worship × Age	–0.001 (–0.002; <–0.001)	<0.001	0.01	
Model 3a Outcome variable: General well-being				0.006
Celebrity worship	–0.01 (–0.02; <0.001)	0.006	0.07	
Opposite/same-gender celebrity worship	0.50 (–0.51; 1.51)	0.51	0.33	
Celebrity worship × Opposite/same-gender	–0.01 (–0.03; 0.01)	0.01	0.30	
Model 3b outcome variable: Self-esteem				0.08
Celebrity worship	–0.05 (–0.07; –0.02)	0.01	<0.001	
Opposite/same-gender celebrity worship	1.08 (–0.89; 3.05)	1.00	0.28	
Celebrity worship × Opposite/same-gender	–0.03 (–0.07; 0.008)	0.02	0.12	
Model 3c outcome variable: Perceived daytime sleepiness				0.03
Celebrity worship	0.03 (0.02; 0.04)	0.006	<0.001	
Opposite/same-gender celebrity worship	–0.25 (–1.28; 0.78)	0.52	0.63	
Celebrity worship × Opposite/same-gender	–0.002 (–0.02; 0.02)	0.01	0.82	

*Age and opposite/same-gender celebrity worship were added as covariates in Model 1a–c, while gender and opposite/same-gender celebrity worship were added as covariates in Model 2a–c, and gender and age were added as covariates in Model 3a–c. For the sake of clarity, covariates are not presented in this table.*

**FIGURE 2:**
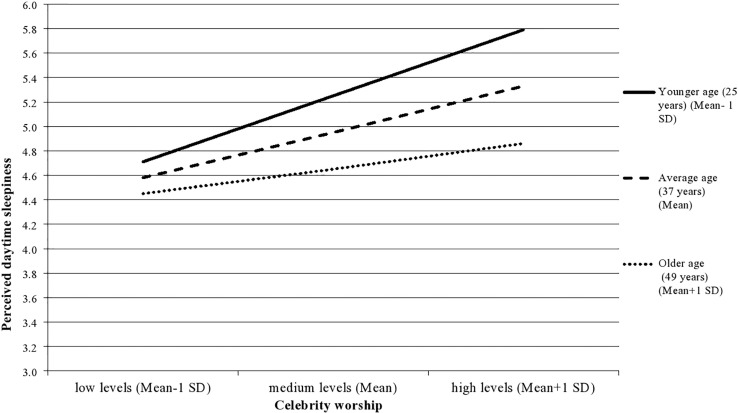
The moderating role of age on the association between celebrity worship and perceived sleepiness.

Supporting the second hypothesis, the moderating role of age was not demonstrated on the association of celebrity worship with general well-being (β < 0.001, *p* = 0.61) and self-esteem (β < 0.001, *p* = 0.24), while the moderating role of age was confirmed on the association between celebrity worship and perceived daytime sleepiness (β = –0.001, *p* = 0.01) (see Model 2a–c in Table 5). This latter association indicates that the relationship between celebrity worship and perceived daytime sleepiness was stronger for younger individuals than for older individuals (see Figure 2). This result suggests that younger individuals with higher levels of celebrity worship are more prone to experience daytime sleepiness than older individuals. Although the interaction term was significant, the moderator effect of age was negligible and the proportion of explained variance was also small (*R*^2^ = 4%). In sum, the second hypothesis received support.

Confirming the third hypothesis, the moderating role of opposite/same-gender celebrity worship was not demonstrated on the association of celebrity worship with general well-being (β = –0.01, *p* = 0.30), self-esteem (β = –0.03, *p* = 0.12), and perceived daytime sleepiness (β = –0.002, *p* = 0.82) (see Model 3a–c in Table 5).

## Discussion

Drawing upon previous findings on the association between celebrity worship and mental health, this study investigated the possible moderator role of gender, age and opposite/same-gender celebrity selection on the association between celebrity worship and some relevant indicators of subjective well-being (i.e., general well-being, self-esteem, and perceived daytime sleepiness) on a relatively large sample of fans. According to the results, gender moderated the association between celebrity worship and self-esteem with a marginal significance level, indicating that women with higher levels of celebrity admiration were slightly more likely to report lower levels of self-esteem than men with an excessive admiration toward a celebrity. Furthermore, the moderating role of age was demonstrated on the association between celebrity worship and perceived daytime sleepiness, indicating that younger individuals with higher levels of celebrity worship experienced more frequent daytime sleepiness compared to older individuals with an excessive fascination with a celebrity. Gender-based selection of a favorite celebrity had no specific role in the association between celebrity worship and subjective well-being. These results indicate that there is a generally weak but consistent negative association between celebrity worship and mental well-being without considerable differences among men and women, younger or older individuals.

Consistent with previous findings (Greenwood and Long, 2011; Swami et al., 2011; Shabahang et al., 2019), the majority of participants (55%) selected a musician/singer or an actor/actress as a favorite celebrity. Furthermore, women were more likely to select a favorite celebrity of the opposite gender when compared with men (73% versus 7%). This result is in line with previous reports (Greenwood et al., 2018; Collisson et al., 2020).

Contrary to the first hypothesis, the moderating role of gender was only demonstrated on the association between celebrity worship and self-esteem, with a marginal significance level. This result indicates that women with an excessive admiration toward a celebrity had slightly lower levels of self-esteem compared to men, suggesting that the attraction toward a glamorous celebrity may have a stronger, negative impact on the self-esteem of women. Although some studies pointed out that an aspirational role model can enhance self-rated attractiveness (Swami et al., 2011) and contribute to the psychological well-being of young women (Lee, 2018) by providing an opportunity to feel socially connected (e.g., online fan communities) (Ang and Chan, 2018) or promoting health behaviors (Hoffman and Tan, 2015), a considerable amount of studies indicated that excessive celebrity worship is associated with body-shape dissatisfaction (e.g., Maltby et al., 2005; Maltby and Day, 2011; Scharf and Levy, 2015), eating disorders (Aruguete et al., 2014; Scharf and Levy, 2015), positive attitudes toward cosmetic surgery (Swami et al., 2009; Jung and Hwang, 2016) and an increased propensity to engage in cosmetic surgery (Maltby and Day, 2011). Indeed, previous research has demonstrated that media exposure to celebrities with “ideal” bodies elevated the risk of body image concerns among women (see King et al., 2000 for a review). Additionally, body shape dissatisfaction was associated with low self-esteem for women but not for men in the study by Furnham et al. (2002). These results may suggest that higher levels of admiration toward a famous person slightly alter the stability of self-esteem in women, while this impact is weaker in men. Future research should investigate which aspects of admiration (e.g., the celebrity’s expertise, physical properties, fame appeal) have a more influential role in the association between celebrity worship and lower self-esteem among men and women. Nevertheless, there is evidence that healthy enthusiasm toward a famous person can also enhance psychological and social well-being (e.g., Ang and Chan, 2018; Lee, 2018, 2019). These findings suggest that an increased interest in a celebrity and excessive worship have differential effects on the psychological well-being of fans, and particular attention should be paid to the level of immersion when exploring fans’ well-being.

In contrast to the expectations, the strength of association of celebrity worship with general well-being and perceived daytime sleepiness was similar for men and women. This result is in line with studies reporting no gender difference in celebrity worship (Ashe et al., 2005; Maltby and Day, 2011), and align with the considerable body of research demonstrating similar levels of subjective well-being in men and women (see Inglehart, 1990 for a review). In relation to perceived daytime sleepiness, Bixler et al. (2005) found no difference in excessive daytime sleepiness with regard to gender, and suggested that multiple factors should be considered when investigating gender differences in daytime sleepiness (e.g., age, health concerns, sleep duration). Therefore, controlling for these factors may have resulted in a more rigorous assessment of gender differences in the strength of association between celebrity worship and perceived daytime sleepiness.

Overall, the present findings suggest that no substantial gender differences could be found in the strength of association between celebrity worship and subjective well-being. Only a small difference was observed in relation to self-esteem, demonstrating that women may be more vulnerable to celebrity influence in this aspect.

Supporting the second hypothesis, age moderated the association between celebrity worship and perceived daytime sleepiness, indicating that younger individuals with higher levels of admiration toward their favorite celebrity report slightly greater daytime sleepiness compared to older individuals. Previous research found evidence for the negative association between age and daytime sleepiness (Bixler et al., 2005; Kim and Young, 2005). Furthermore, it was found that celebrity worship slightly decreased with age (see Brooks, 2018 for a review). The present findings provide further support for these associations. One possible explanation for these results could be that individuals with an excessive admiration toward a celebrity may spend more time daydreaming, which has been associated with daytime sleepiness (Carciofo et al., 2014). Supporting this notion, younger individuals reported more maladaptive daydreaming experiences (Bigelsen et al., 2016), and maladaptive daydreaming tendencies were associated with higher levels of celebrity worship (Zsila et al., 2018). Additionally, both maladaptive daydreaming and celebrity worship were associated with symptoms of depression and anxiety (Sansone and Sansone, 2014; Ross et al., 2020), which were found to be related to sleep disturbances (see Alvaro et al., 2013 for a review). Provided that daydreaming fantasies were reported occurring before sleep in most cases, these experiences can affect sleep quality (Schimmenti et al., 2019), which in turn can increase the sense of daytime sleepiness (Carciofo et al., 2014). Future research should investigate the role of maladaptive daydreaming in the relationship between celebrity worship and perceived daytime sleepiness in more depth.

In support of the third hypothesis, no evidence was found for the moderating role of opposite- or same-gender celebrity selection on the association between celebrity worship and subjective well-being. Recent studies addressed the question of whether fans differ in their admiration levels based upon opposite- or same-gender celebrity selection (Greenwood et al., 2018; Collisson et al., 2020). While Greenwood et al. (2018) found higher levels of celebrity worship among students with a favorite celebrity of the opposite gender, Collisson et al. (2020) found no difference in the level of admiration in a sample of adults. The present findings align with the results reported by Collisson et al. (2020), who suggested that the lack of difference in celebrity worship levels for opposite- and same-gender celebrity worshippers may be due to the differential role of a celebrity in individuals’ life. As was proposed by Collisson et al. (2020), individuals selecting a favorite celebrity of the same gender may consider the celebrity as a role model, and the identification with him/her increases their fascination with the person who represents the ideal self of the fans, which in turn enhances fans’ obsessive feelings and dedication for the celebrity. Further supporting this assumption, previous studies have indicated that similarity with a famous person based on major demographic characteristics (e.g., gender, race) can intensify the emotional attachment to the celebrity (Klimmt et al., 2006; Giuliano et al., 2007). Findings of the present study also suggest that a favorite celebrity may have a differential role in the life of fans; therefore, the emotional function of the parasocial relationship (i.e., one-sided emotional bond with a famous person as defined by Giles, 2002) can possibly determine its influence on psychological well-being.

The present study has a number of limitations that need to be considered when interpreting the results. First, general conclusions for the whole population of Hungarian adults cannot be drawn from the present findings. The convenience sampling method applied for the present data collection limits the generalizability of the results. Therefore, the associations presented in this study may not reflect the behavioral mechanisms observed in the general population accurately. Representative samples are needed to confirm the present findings. Moreover, data were gathered from one website, which can further limit the generalizability of the findings. Future studies should conduct a broader data collection procedure. Second, the direction of association between celebrity worship and subjective well-being cannot be ascertained due to the cross-sectional study design. It is possible that higher levels of celebrity worship deteriorate psychological functioning, but it is also plausible that poorer mental health increases the risk of developing an obsessive emotional bond to a famous person. To draw clearer conclusions on directionality, longitudinal research is needed. Third, only a few aspects of well-being were examined in relation to celebrity worship in the present study. Future studies should investigate more aspects of subjective well-being (e.g., positive and negative affect, happiness, physical health) to gain a more nuanced picture of the relationship between celebrity worship and well-being. Future research should also test the possible moderating role of other relevant factors associated with celebrity worship (e.g., social connectedness, coping styles). Additionally, the moderator effect of gender and age was small in the present study. Further research is needed to confirm the moderating role of these demographic characteristics. For this purpose, more diverse samples should be used in terms of gender and age. Another limitation of the present study is that the assessment instruments assessing celebrity worship and perceived daytime sleepiness have not yet been validated for Hungarian adults. Therefore, it is plausible that the meaning of some items slightly varies in this cultural context, which can possibly bias the results. Finally, future studies that use more multidimensional constructs and produce more powerful moderating effects could possibly benefit from using structural equation modeling (SEM) techniques that enable the exploration of the strength of moderating effects in a comprehensive model.

Overall, the present study found evidence for a small moderating effect of gender and age on the association between celebrity worship and some indicators of psychological well-being (i.e., self-esteem and perceived daytime sleepiness). These results point out that (1) female self-esteem may be slightly more vulnerable to celebrity influence, (2) younger individuals may be slightly more prone to feel sleepy during the day when they excessively admire a famous person. Previous studies have consistently found a weak, negative association between celebrity worship and mental well-being (see Sansone and Sansone, 2014 for a review); however, studies investigating possible individual differences in this association are scarce. This study offered a more nuanced picture of the nature of this association by drawing attention to some demographic characteristics that can possibly alter the strength of the relationship between celebrity admiration and subjective well-being. The present investigation provided further evidence for the weak, negative association between celebrity worship and psychological well-being, and pointed out that there are no substantial differences in the strength of this association across gender and different age groups. This result suggests that excessive levels of celebrity admiration may have similar consequences to mental health, irrespective of gender and age, although some specific subgroups (i.e., women and younger individuals who admire a celebrity) may need particular attention when experiencing high levels of celebrity worship. In order to avoid adverse effect of an obsessive admiration toward a favorite celebrity, more effective harm-reducing strategies could be used for these specific groups based on the present results. For instance, particular attention should be paid to enhance the self-esteem of highly engaged women to prevent them from developing severe mental health problems in the level of self-concept. Indeed, women with high levels of celebrity worship were found to be more prone to report lower levels of self-esteem than men in the present study. Although the difference was small, this result point out that enhancing female fans’ self-esteem may be important to prevent them from developing more severe mental health concerns. Furthermore, self-regulatory strategies to control the time spent fantasizing about an admired celebrity may be more effective for younger fans to prevent them from experiencing excessive daytime sleepiness. Drawing on the present results, younger fans may indeed benefit from controlling the time spent daydreaming about a favorite celebrity in order to avoid the feelings of being exhausted and sleepy during the day, which can possibly affect cognitive performance.

Overall, the findings of the present study can contribute to the growing body of literature on the relationship between celebrity worship and psychological well-being, and extend previous knowledge on the possible individual differences that can alter the strength of this association. Provided that the group differences were particularly small in this study, future research is needed to confirm these differences. Furthermore, the investigation should be extended to other possible contributing factors such as loneliness, social support and personality traits. The exploration of these social and personality characteristics could increase knowledge about the nature of the association between celebrity worship and psychological well-being.

## Data Availability Statement

The datasets generated for this study are available on request to the corresponding author.

## Ethics Statement

The studies involving human participants were reviewed and approved by Institutional Review Board of ELTE Eötvös Loránd University. The patients/participants provided their written informed consent to participate in this study.

## Author Contributions

ÁZ: conceptualization and writing the first draft. GO: formal analysis and writing and editing the draft. LM: conceptualization and writing and editing the draft. ZD: supervision and writing and editing the draft. All authors contributed to the article and approved the submitted version.

## Conflict of Interest

The authors declare that the research was conducted in the absence of any commercial or financial relationships that could be construed as a potential conflict of interest.

## Footnotes

1 444.hu
